# Landscape of Carbon Ion Radiotherapy in Prostate Cancer: Clinical Application and Translational Research

**DOI:** 10.3389/fonc.2021.760752

**Published:** 2021-11-05

**Authors:** Xue Chen, Qi Yu, Ping Li, Shen Fu

**Affiliations:** ^1^ Department of Radiation Oncology, Fudan University Shanghai Cancer Center, Shanghai, China; ^2^ Department of Oncology, Shanghai Medical College of Fudan University, Shanghai, China; ^3^ Proton & Heavy Ion Medical Center, State Key Laboratory of Radiation Medicine and Protection, School of Radiation Medicine and Protection, Soochow University, Suzhou, China; ^4^ Department of Radiation Oncology, Shanghai Concord Cancer Center, Shanghai, China; ^5^ Department of Radiation Oncology, Shanghai Proton and Heavy lon Center, Shanghai, China; ^6^ Key Laboratory of Nuclear Physics and Ion-beam Application (MOE), Fudan University, Shanghai, China

**Keywords:** carbon ion radiotherapy, efficacy, prostate cancer, quality of life, translational research

## Abstract

Carbon ion radiotherapy (CIRT) is a useful and advanced technique for prostate cancer. This study sought to investigate the clinical efficacy and translational research for prostate cancer with carbon ion radiotherapy. We integrated the data from published articles, clinical trials websites, and our data. The efficacy of CIRT for prostate cancer was assessed in terms of overall survival, biochemical recurrence-free survival, and toxicity response. Up to now, clinical treatment of carbon ion radiotherapy has been carried in only five countries. We found that carbon ion radiotherapy induced little genitourinary and gastrointestinal toxicity when used for prostate cancer treatment. To some extent, it led to improved outcomes in overall survival, biochemical recurrence-free survival than conventional radiotherapy, especially for high-risk prostate cancer. Carbon ion radiotherapy brought clinical benefits for prostate cancer patients, and quality of life assessment indicated that CIRT affected patients to a lesser extent. Potential biomarkers from our omics-based study could be used to predict the efficacy of prostate cancer with CIRT. Carbon ion radiotherapy brought clinical benefits for prostate cancer patients. The omics-based translational research may provide insights into individualized therapy.

## Introduction

Prostate cancer is a common malignancy in Europe and the United States, ranking first and second in incidence and mortality rates, respectively (1). The incidence rate of prostate cancer in the United States in 2012 was documented to be 20 times higher than in Asia, yet the mortality rate is only 2.5 times superior (2, 3); The National Cancer Center of China reported that from 2000-2014, the incidence of prostate cancer in China increased year by year, from 4.62/100,000 to 21.62/100,000, with a higher incidence in urban than in rural areas, while the incidence of patients older than 65 years accounted for more than 80% (4). Given the large difference in the efficacy of prostate cancer treatment in China and western countries (5, 6), it is worth exploring how current treatment approaches could be improved to improve treatment efficacy.

The selection of treatment approaches for prostate cancer therapy is usually based on the clinical stage; unfavorable intermediate-risk and high-risk prostate cancer are currently treated with a comprehensive approach, combining surgical treatment, radiotherapy, and endocrine therapy. Radiotherapy has become a major therapeutic option for prostate cancer, recommended by NCCN guidelines for localized and locally advanced prostate cancer (7). Men with low-risk and intermediate-risk favorable prostate cancer who have a life expectancy of at least 10 years can be managed with active surveillance, radical prostatectomy, external beam radiotherapy or brachytherapy. The common radiotherapy modalities for prostate cancer include external beam radiotherapy and brachytherapy or a combination of both (8). External beam radiotherapy mainly includes intensity-modulated radiation therapy (IMRT), image-guided intensity-modulated radiation therapy (IGRT) and stereotactic radiation therapy (SBRT) (9), volumetric modulated arc therapy (VMAT) (10); brachytherapy includes low-dose-rate and high-dose-rate brachytherapy (11). The conventional irradiation beam is photon; however, with the progress made in biomedical research, protons and carbon ions are nowadays used for clinical treatments. Conventional photon radiotherapy with a dose range of 70-80 Gy, and radiotherapy combined with endocrine therapy has been documented to improve patients survival in the intermediate-risk and high-risk group (12). Clinically, a dose of more than 80 Gy is usually used to boost clinical target volume or an intraprostatic lesion (IPL) in prostate cancer; for example, Fonteyne et al. performed a simultaneous integrated boost with a median dose of 81 Gy and 82 Gy to an IPL detected by magnetic resonance imaging with or without spectroscopy, and it did not increase acute toxicity rate (13).

Conventional irradiation is given in fractionated doses of 1.8-2.0 Gy. Interestingly, studies have shown that hypofractionated irradiation is not inferior to conventional irradiation for prostate cancer. CHHiP was a phase 3 trial where patients were randomized to one of three dose schedules: 74 Gy/37 fractions (1065 patients) in conventional irradiation group, 60 Gy/20 fractions (1074 patients) and 57 Gy/19 fractions (1077 patients) in hypofractionated irradiation group, with a median follow-up of 62.4 months; the primary study endpoint is biochemical recurrence or clinical recurrence. The 5-year biochemical or clinical recurrence-free rates were 90.6% (60 Gy) and 85.9% (57 Gy) in the hypofractionated radiotherapy group, and 88.3% (74 Gy) in the conventional irradiation group; long-term toxicity effects were comparable after hypofractionated and conventional radiotherapy (14). Moreover, toxicity rates with 57 Gy/19 fractions were inferior to 60 Gy/20 fractions and conventional fractionation, suggesting that hypofractionated radiation therapy’s efficacy and toxicity reactions were within an acceptable range. However, patients in that study were predominantly intermediate-risk patients, with 779 (73%) intermediate-risk patients in the conventional irradiation group and 784 (73%) intermediate-risk patients in the hypofractionated irradiation group. Actually, a significant portion of patients are diagnosed with high-risk prostate cancer, and disease control is often challenging in such cases (15, 16). Accordingly, new approaches should be explored to improve the outcomes of this patient population. High-dose radiation has huge prospects for achieving disease control in high-risk prostate cancer. Studies have shown that high-dose irradiation further improved local control and biochemical recurrence-free survival (bRFS); however, genitourinary toxicity increased at high-dose radiotherapy such as 70 Gy *versus* 80 Gy (17). Therefore, it is crucial to balance tumor control and toxicity response in prostate cancer patients receiving carbon ion radiotherapy.

Researchers in Japan, Germany and the United States have clarified the feasibility of proton and heavy-ion radiotherapy during clinical practice; the clinical application of proton and heavy ions, especially *via* carbon ion radiotherapy, provides new directions for prostate cancer treatment (18–20). Carbon ions offer both physical and biological advantages (21). In terms of physical advantage, by delivering a spread-out Bragg peak proportional to the tumor size, the incident dose of carbon ion is deposited less on the skin surface with most of the energy deposited within the tumor target area and directed to blast and kill the tumor cells, with almost no outgoing dose deposited in normal tissue (22). Compared to proton beams, carbon ion beams exhibit less coulomb scattering and sharper lateral penumbra (23). In terms of biological effects, the linear energy transfer (LET) of carbon ions is high, and the relative biological effect (RBE) of carbon ions is higher than that of photon and proton, which is usually 1 for photon and about 1.1 for proton, while the RBE of carbon ions can be 2 to 3 or higher (24). Carbon ions have a low oxygen enhancement ratio and killing tumor cells does not require oxygen radicals to damage tumor cells’ DNA, so carbon ions can be harnessed to kill anoxic cells. Furthermore, CIRT, unlike photon radiotherapy, is not cell cycle dependent (23, 25).

Carbon ion radiotherapy could be used to kill prostate cancer cells while mitigating normal tissue damage, especially for surrounding organs at risk, such as the rectum and bladder; much emphasis should be laid on dose optimization. Indeed, increasing current knowledge on the clinical application of carbon ion radiotherapy warrants further study (26). This study investigated the application of carbon ion radiotherapy in prostate cancer in terms of clinical efficacy, toxicity response, and quality of life assessment.

## Methods and Materials

### Data Extraction and Objects

We used the following keywords: prostate carcinoma, particle therapy, carbon ion radiotherapy, quality of life, toxicity response, clinical efficacy, clinical outcome, biochemical control, biochemical recurrence-free survival, bRFS to search relative articles published by English in PubMed, Medline and Web of Science. We mainly focused on clinical research and translational research based on omics.

### Statistics of Patients Treated in Carbon Ion Radiotherapy

The data came from the official website of PTCOG (Particle Therapy Co-Operative Group), which included the number of institutions and patients who received carbon ion radiotherapy. The clinical trials were extracted from the website of the Clinical Trials Registry (https://clinicaltrials.gov/) and PTCOG.

### Assessment of Efficacy and Toxicity

Clinical outcomes including prostate cancer-specific survival (PCSS), biochemical recurrence-free survival (bRFS), and overall survival (OS) were evaluated. Gastrointestinal (GI) and genitourinary (GU) acute and late toxicities were assessed according to Common Terminology Criteria for Adverse Events v4.03 (CTCAE v4.03) and Radiation Therapy Oncology Group (RTOG)/European Organization for Research and Treatment of Cancer (EORTC), respectively (27, 28). The quality of life was assessed by functional assessment of cancer therapy (FACT) and trail outcome index (TOI), European Organization for Research and Treatment of Cancer (EORTC) QLQ-C30 and QLQ-PR25 questionnaires, Expanded Prostate Cancer Index-26 (EPIC-26) in Japan, Germany, China, respectively.

## Results

### Patient Statistics of Carbon Ion Therapy Facilities Worldwide

To date, more than 30,000 patients worldwide have been treated with carbon ion radiotherapy, according to PTCOG. Five countries, including Japan, Germany, China, Italy and Austria operate carbon ion radiotherapy facilities; **Table 1** summarizes the institutions worldwide where carbon ion radiotherapy has been performed. Three institutes perform carbon ion therapy in China, among which the Shanghai Proton and Heavy Ion Center (SPHIC) has treated more than two thousand patients. German centers including the German Gesellschaft fur Schwerioneforschung (GSI), the Heidelberg Ion Therapy Center (HIT), and Marburger Ionenstrahl-Therapiezentrum (MIT) are also widely recognized for carbon ion therapy (29). The National Institute of Radiological Sciences (NIRS) was the first center in Japan to treat tumors with carbon ions since 1994 (30). Then Hyogo Ion Center (HIBMC), Gunma Heavy Ion Center (GHMC), Saga Heavy Ion Center (Saga-HIMAT), Kanagawa Heavy Ion Center (i-Rock), Osaka Heavy Ion Therapy Center in Japan started to treat patients with carbon ion therapy (31, 32). The CNAO Proton Heavy Ion Therapy Center and the Wiener Neustadt (MedAustron) in Austria started to treat patients in 2012 and 2019, respectively.

**Table 1:** Institutions perform carbon ion radiotherapy (update to 2020).

Country	Site	Particle	Start time	Patients total	Deadline
China	Lanzhou	C-ion	2006	213	19-Dec
China	Shanghai (SPHIC)	C-ion	2014	2249	19-Dec
China	Wuwei	C-ion	2019	46	19-Dec
Germany	Darmstadt (GSI)	C-ion	1997 (-2009)	440	2009
Germany	HIT, Heidelberg	C-ion	2009, 2012	3468	19-Dec
Germany	MIT, Marburg	C-ion	2015	322	18-Dec
Italy	Pavia (CNAO)	C-ion	2012	1534	19-Dec
Japan	Chiba (HIMAC)	C-ion	1994, 2017	13489	19-Dec
Japan	Hyogo (HIBMC)	C-ion	2002	3037	19-Dec
Japan	Gunma (GHMC)	C-ion	2010	3821	19-Dec
Japan	Tosu (Saga-HIMAT)	C-ion	2013	2583	18-Mar
Japan	Kanagawa (i-Rock)	C-ion	2015	989	19-Dec
Japan	Osaka Heavy Ion Therapy Center	C-ion	2018	First patient	18-Oct
Austria	Wiener Neustadt (MedAustron)	C-ion	2019	22	19-Dec

C-ion, carbon ion; SPHIC, Shanghai Proton and Heavy Ion Center; GSI, Gesellschaft fur Schwerioneforschung; HIT, Heidelberg Ion Therapy Centre; HIBMC, Hyogo Ion Center; GHMC, Gunma Heavy Ion Center; Saga-HIMAT, Saga Heavy Ion Center; i-Rock, Kanagawa Heavy Ion Center.

### Clinical Trials of Carbon Ion Radiotherapy for Prostate Cancer

Clinical trials of carbon ion radiotherapy for prostate cancer were retrieved from the official website of the Clinical Trials Registry (https://clinicaltrials.gov/) and PTCOG (Particle Therapy Co-Operative Group). Currently, there are clinical trials of carbon ion radiotherapy for prostate cancer in China, Italy, Germany, and Japan (**Table 2**). With two ongoing clinical trials for prostate cancer at the Shanghai Proton and Heavy Ion Center (SPHIC).

**Table 2:** Clinical trials of prostate cancer with carbon ion radiotherapy.

Institute	Trial number	Conditions	Interventions	Primary end-point
SPHIC/China	NCT02739659	Localized prostate cancer	Carbon ion	Toxicity
SPHIC/China	NCT02935023	Oligo-metastatic prostate cancer	CIRT plus systemic therapy	bRFS
IEO/Italy	NCT02672449	High risk prostate cancer	Carbon ion boost plus pelvic Photon RT	Toxicity
HIT/Germany	NCT01641185	Localized prostate cancer	CIRT or PRT	Toxicity
NIRS/Japan	JCROS-1509	High-risk prostate cancer	CIRT plus hormone therapy	5-year bRFS
iROCK/Japan	iROCK-1501PR	Prostate cancer, T1c-T3N0M0	Carbon ion RT	5-year bRFS

SPHIC, Shanghai Proton and Heavy Ion Center; IEO, European Institute of Oncology; HIT, Heidelberg Ion Therapy Centre; NIRS, National Institute of Radiological Sciences; iROCK, Ion Beam Radiation Oncology Center in Kanagawa; CIRT, carbon ion radiotherapy; RT, radiotherapy; bRFS, Biochemical Recurrence-free Survival.

### Clinical Efficacy of Carbon Ion Radiotherapy for Prostate Cancer

#### Experience With Carbon Ion Radiotherapy in NIRS

The NIRS has been using carbon ions to treat tumors since 1994 in Japan, conducted the first dose-escalation study of carbon ion therapy for prostate cancer between July 1995 and December 1997 (protocol 9402) to determine the optimal dose of carbon ion therapy (33, 34). The total dose was increased from 54 GyE to 72 GyE with 20 fractions/5 weeks. The median follow-up time was 47 months. Local control was achieved in all patients except one patient irradiated with 54 GyE, but five of the 14 patients (36%) irradiated with 72 GyE developed late grade 3 toxicities involving the rectum and bladder/urethra. Accordingly, the 72 GyE dose was subsequently discontinued and replaced by a 66 GyE irradiation regimen. The results of this clinical trial were used to determine the maximum tolerated dose by the rectum in carbon ion radiotherapy and determine the appropriate dose range for carbon ion therapy for prostate cancer. The overall survival rate, cause-specific survival rate, bRFS, and local control was 87.7%, 94.9%, 82.6%, 98.5%, respectively (**Table 3**).

**Table 3:** Clinical outcome in prostate cancer patients treated with carbon ion radiotherapy.

Authors	Year	No. of patients	Radiation therapy	Median of follow-up	Clinical outcome
Akakura et al. (34)	2004	96	Carbon ion	47 months	5-year OS: 87.7%; cause-specific survival: 94.9%; clinical recurrence-free survival: 90%; bRFS: 82.6%; local control: 98.5%
			54-72 GyE/20 fractions		No grade 3 or worse acute toxicity
					Grade 1, 2, 3 late GI toxicity: 12, 6, 4 cases, respectively
					Grade 1, 2, 3 late GU toxicity: 28, 5, 6 cases, respectively
Tsuji et al. (35)	2005	201	Carbon ion	NA	5-year OS 89.2%; bRFS 83.2%; local control 100%
			54-72 GyE/20 fractions		Grade 2 GI and GU toxicity: 1% and 6%
			66 GyE/20 fractions		No Grade 3 or higher GI and GU toxicities
Ishikawa et al. (36)	2006	175	Carbon ion	46 months	4-year OS and bRFS: 91% and 87%
			66 GyE/20 fractions		4-year bRFS in low-risk and high-risk group: 87% and 88%
					Grade 2 late GI and GU toxicity: 2% and 5%
					No Grade 3 or higher toxicities
Okada et al. (37)	2012	740	Carbon ion	59.3 months	5-year OS and bRFS: 95.2% and 89.7%
			63 or 66 GyE /20 fractions		Grade 1 and 2 late GI toxicity: 14% and 2.4%
			57.6GyE/16 fractions		Grade 1, 2 and 3 late GU toxicity: 43.1%, 7.8% and 0.2%
					GU toxicity with 57.6 GyE/16 fractions was lower than 63 or 66 GyE/20 fractions
Nomiya et al. (38)	2014	46	Carbon ion	32.3 months	Grade1 late toxicity of rectal hemorrhage: 7%
			51.6GyE/12 fractions		Grade1 late toxicity of hematuria: 13%
					Grade1late toxicity of urinary frequency: 37%
					No ≥Grade 2 late toxicities
					Grade2 acute toxicity of urinary frequency: 4%
					No other grade 2 acute toxicities
Nomiya et al. (39)	2016	2157	Carbon ion	43 months in NIRS	5-year bRFS in low-, intermediate-, and high-risk patients: 92%, 89%, 92%
			63 or 66 GyE/20 fractions	23 months in GHMC	5-year CSS in low-, intermediate-, and high-risk patients: 100%, 100%, 99%
			57.6GyE/16 fractions	7 months in HIMAT	5-year LCR in low-, intermediate-, and high-risk patients: 98%, 96%, 99%
			51.6GyE/12 fractions	29 months in all surviving patients	5-year OS in low-, intermediate-, and high-risk patients: 100%, 99%, 96%
					Grade 2 late GU and GI toxicities: 4.6% and 0.4%
					No Grade 3 or higher late toxicities
Habl et al. (40)	2016	91	Proton (arm A, n=46)	22.3 months	Grade 1 cystitis: 34.1% (39.1% in A; 28.9% in B)
			Carbon ion (arm B, n=45)		Grade 2 cystitis: 17.6% (21.7% in A; 13.3% in B)
			66 GyE/20 fractions		Grade 1 radiation proctitis: 12.1% (13.0% in A; 11.1% in B)
					Grade 2 radiation proctitis: 5.5% (8.7% in A; 2.2% in B).
					Grade 3 radiation proctitis: 2.2% (4.3% in A; 0% in B)
					Grade 1 diarrhea: 58.2% (60.9% in A; 55.6% in B)
					Grade 2 diarrhea: 4.4% (8.7% in A; 0% in B)
					
Kasuya et al. (41)	2017	608	Carbon ion	88.4 months	5-/10-year PCa-specific mortality rates were 1.5%/4.3%
			63 or 66 GyE /20 fractions		
			57.6 GyE/16 fractions		
					
Zhang et al. (42)	2019	64	Carbon ion	19 months	Grade 1 acute GU toxicity: 20.3%
			59.2-60.8 GyE /16 fractions		Grade 2 acute GU toxicity: 10.9%
			66 GyE/24 fractions		Grade 1 late GU toxicity: 3.1%
					Grade 2 late GU toxicity: 1.6%
					No acute or late GI toxicity
					
Takakusagi et al. (43)	2020	253	Carbon ion	35.3 months	3-year bRFS in low-, intermediate-, and high-risk patients: 87.5%, 88%, 97.5%
			51.6GyE/12 fractions		Grade 2 acute urinary toxicity: 4.7%
					No grade 2 acute rectal toxicity
					Grade 2 late GU and GI: 6.7% and 1.2%
					
Kawamura et al. (44)	2020	304	Carbon ion	60 months	5-year bRFS, OS, local control: 92.7%, 96.6%, 98.4%
			57.6GyE/16 fractions		5-year bRFS in low-, intermediate-, and high-risk patients: 91.7%, 93.4%, 92%
					Late grade 2 GU and GI: 9% and 0.3%
					Late grade 3 GU and GI: 0.3% and 0%
					No grade 3 or great acute toxicity
					
Sato et al. (45)	2021	256	Carbon ion	7 years	5-year bRFS in low-, intermediate-, and high-risk patients: 95.1%, 90.9%, 91.1%
			51.6GyE/12 fractions		Late grade 2 GU and GI: 6.3% and 0.4%
					No grade 3 or higher toxicities

OS, overall survival; bRFS, biochemical recurrence-free survival; CSS, cause-specific survival; LCR, local control rate; GI, Gastrointestinal; GU, genitourinary; NA, not available; NIRS, National Institute of Radiological Sciences; GHMC, Gunma University Heavy Ion Medical Center; HIMAT, Ion Beam Therapy Center, SAGA HIMAT Foundation.

Next, they conducted a series of clinical trials to explore the best regimen for prostate cancer patients. A phase II clinical initiated in January 1998 (protocol 9703) using the reduced field irradiation technique for localized and advanced prostate cancer (34). The study was completed in March 2000 and no grade 3 late toxicity was reported. Based on the above findings, the investigators designed a phase II clinical study (protocol 9904) and a 66 GyE/20Fr schedule was adopted to further confirm the efficacy of carbon ion radiation therapy in patients with stage T1-T3 prostate cancer (N=176). Local control was achieved in all but one patient, and grade 2 GI and GU toxicities were observed in 2% and 5% of patients, respectively.

The HAMT clinical trial was conducted in November 2003 and continued with the administration of 66 GyE/20Fr irradiation to treat 120 patients. Since the beginning of the HAMT trials in 2003, 246 patients were given a dose of 57.6 GyE/16Fr (protocol 0507), which shortened the treatment time from 5 to 4 weeks (37). This study clarified that carbon ion radiotherapy is safe and effective when given at 57.6 GyE/16Fr. A clinical trial with a 3-week irradiation protocol of 51.2 GyE was conducted in 2010 (protocol 1002) (38), the median follow-up time was 32.3 months; the study confirmed that the acute toxicity rate of the rectum and bladder/urethra was acceptable. Grade 2 GU acute toxicity reactions were found in 4% of patients irradiated with 51.2 GyE/12Fx (n=49), and no grade 2 GI acute toxicity reactions were observed. Therefore, a dose of 51.2 GyE/12Fx was prescribed for subsequent NIRS. The clinical outcome of 51.2 GyE/12Fx regimen indicated that 5-year bRFS in low-, intermediate-, and high-risk patients was 95.1%, 90.9%, 91.1%, respectively; and the late grade 2 GU and GI toxicity was 6.3% and 0.4% (45). Studies on carbon ion radiotherapy for prostate cancer at other institutions in Japan are shown in **Table 3**.

Late toxicities were analyzed in 250 patients irradiated with 66 GyE/20Fr, 216 patients with 63 GyE/20Fr, and 461 patients with 57.6 GyE/16Fr. The median follow-up period for all 927 patients was 43 (6-133) months. Grade 2 toxicity presenting as rectal bleeding was observed in 15 (1.6%) cases, but no grade 3 or more severe rectal toxicity reactions were observed in all groups. Late grade 2 and grade 3 GU toxicity reactions were observed in 57 (6.1%) and 1 (0.1%) of the 927 patients, respectively, and most toxicity reactions were attenuated or resolved at the last follow-up. 5-year prostate cancer-specific survival was 98.8%, 5-year OS was 95.3%, 5-year local control was 98.3% and 5-year bRFS was 90.6%.

#### Experience of Multicenter Carbon Ion Radiotherapy in Japan

Heavy-ion has been developed over the years, with five institutions carrying out carbon ion radiotherapy in Japan. In 2016, NIRS, in conjunction with Gunma Heavy Ion Center (GHMC) and Saga Heavy Ion Center (HIMAT) published a multicenter study (J-CROS1501PR) (39). The study included a total of 2157 patients with irradiation regimens of 66 GyE/20fr-51.6 GyE/12fr between 2000 and December 2014. According to the D’Amico risk classification, low-risk, intermediate-risk and high-risk patients accounted for 12.2%, 31.5% and 56.3%, respectively. Low-risk patients were given carbon ion radiotherapy alone; intermediate-risk patients received 4-8 months of neoadjuvant endocrine therapy, and high-risk patients received 24 months of neoadjuvant and adjuvant endocrine therapy. The median follow-up time in this study was 43, 23, and 7 months at NIRS, GHMC, and HIMAT, respectively. The 5-year bRFS was 92%, 89%, 92% (p=0.22), 5-year prostate cancer-specific survival (PCSS) was 100%, 100%, 99% (p=0.42), and 5-year OS was 100%, 99%, 96% (p=0.0546) in low-risk, intermediate-risk, and high-risk patients, respectively. Grade 2 GU and GI toxicities were 4.6% and 0.4%, respectively, and no grade 3 or higher toxicities. The results suggest that carbon ion radiotherapy for prostate cancer is safe effective, and beneficial for patients with high-risk prostate cancer. Therefore, this non-invasive and short-course carbon ion radiotherapy is worthy of further promotion in the treatment of prostate cancer.

#### Experience With Carbon Ion Radiotherapy for Prostate Cancer in Germany

The IPI clinical trial (40) was conducted at the Heidelberg Heavy Ion Center in Germany in 2012, enrolling 92 patients with localized prostate cancer to compare the safety and feasibility of proton and carbon ion radiotherapy using a raster scan beam. Finally, 91 patients were randomized to the proton radiotherapy group (n=46) and the carbon ion radiotherapy group (n=45), and they were given 66 GyE/20 Fr irradiation. The median follow-up time was 22.3 months, and the GU toxicity was 39.1% in the proton group for grade 1 cystitis, 28.9% in the carbon ion group; 21.7% in the proton group for grade 2 cystitis, and 13.3% in the carbon ion group; GI toxic reactions were 13% in the proton group and 11.1% in the carbon ion group for grade 1 of proctitis, 8.7% in the proton group and 2.2% in the carbon ion treatment group for grade 2 of proctitis. Two patients developed grade 3 rectal fistula after proton therapy, which may be related to spacer gel in the rectum, so the gel was discontinued in the follow-up study.

#### Experience With Carbon Ion Radiotherapy for Prostate Cancer in Italy

A clinical trial (NCT02672449) of mix-beam (carbon ions and photons) radiotherapy for high-risk prostate cancer was conducted in Italy’s European Institute of oncology. They estimated to enroll 65 patients received carbon ion boost followed by pelvic photon radiotherapy, and the prostate boost with carbon ions will be 16.6 GyE in 4 fractions; they aimed to improve the current treatment for high-risk prostate cancer and evaluate the safety and feasibility of mix-beam radiotherapy for high-risk prostate cancer (46). To clarify the potential power of this mix-beam irradiation, they retrospectively analyzed the data of 76 patients treated with photon radiotherapy (47); After a median follow-up of 20.2 (5-58.1) months, 22 (28.9%) patients had biochemical progression, and 16 patients (21.1%) had clinical progression. These results indicated that a more aggressive treatment was necessary for high-risk prostate cancer. They believed that carbon ion radiotherapy combined with photon radiotherapy for high-risk prostate cancer is a promising strategy. However, the results of this mix-beam radiotherapy have not been reported. The different studies on carbon ion radiation therapy for prostate cancer showed in **Table 3**.

#### Factors Related to the Effect of Carbon Ion Radiotherapy

A previous Japanese study found that gastrointestinal toxicity after carbon ion radiotherapy for prostate cancer was dominated by rectal bleeding, and the Ishikawa al. found that rectal bleeding was mainly associated with rectal V50 dose (48) and whether anticoagulant drugs were taken (49).

Biochemical recurrence of prostate cancer is an important factor affecting prognosis. Shimazaki et al. explored the main factors of poor prognosis due to biochemical recurrence after carbon ion radiotherapy for prostate cancer and found that high tumor pathological grade and a short time to biochemical recurrence were associated with poor prognosis (50). Kasuya et al. found that biochemical recurrence was an independent prognostic factor for overall mortality, independent of the duration of endocrine therapy (51), by retrospectively analyzing 466 cases of prostate cancer receiving 63-66 GyE from 2000 to 2007, of which there were 324 cases of high-risk prostate cancer (52).

### Quality of Life Assessment of Carbon Ion Radiotherapy for Prostate Cancer

NIRS published a study in 2017 to assess the quality of life for prostate cancer with carbon ion radiotherapy (53). The study included 417 patients who received 63-66 GyE/20Fr irradiation, with neoadjuvant and adjuvant endocrine therapy to moderate and high-risk patients. Quality of life was assessed at five-time points: before radiotherapy, after radiotherapy, 12 months after radiotherapy, 36 months after radiotherapy, and 60 months after radiotherapy. The scores were collected mainly by questionnaire using the Functional Assessment of Cancer Therapy (FACT) scale. The study results showed that the FACT-G and FACT-P scores were significantly lower, but the difference was not significant after 60 months of treatment. The Trial Outcome Index (TOI) scores decreased briefly and then gradually returned to baseline; patients treated with endocrine therapy had a high probability of adverse effects and patients with biochemical relapse had lower quality-of-life scores. Overall, patients’ quality of life treated with carbon ion radiotherapy was like that of other treatment modalities. Follow-up is needed to validate the results of this study in a larger population.

The quality of life assessment of the German IPI clinical trial used the EORTC QLQ-C30 and PR25 questionnaires to collect scores on all scales (40). Functional scores were low for all scales except cognitive and emotional functioning, dyspnea, insomnia, and financial difficulties, and higher scores for symptom-related scales, suggesting that the quality of life associated with radiotherapy was compromised during radiotherapy. All scores increased during follow-up, suggesting a gradual improvement in quality of life. Urologic symptoms, fatigue, and pain decreased at the end of treatment and increased after six months of follow-up for urologic symptoms and fatigue, but fatigue did not improve. PR25 symptom scores revealed significant differences in urethral (p=0.026) and rectal (p=0.046) symptoms between the proton therapy and carbon ion therapy groups. Bowel scores were significantly higher at the end of treatment than before treatment but improved significantly after six weeks of follow-up (p=0.046). The proton and carbon ion groups reached their initial pre-treatment levels of sexual performance at six weeks after treatment and did not experience significant sexual dysfunction. This shows that the quality of life of patients with prostate cancer treated with carbon ion and proton was slightly affected during the treatment period but improved at the end of treatment in both cases.

Shanghai Proton and Heavy Ion Center conducted a study on quality of life assessment and toxicity reactions in prostate cancer patients treated with carbon ions (42). Sixty-four patients were included, 18 patients received 64 GyE/24Fr irradiation and 46 patients were given 59.2-60.8 GyE/16Fr irradiation, and urinary, bowel and sexual function were assessed by the Expanded Prostate Cancer Index-26 (EPIC-26, 26-item edition of the Composite) to assess urinary tract, bowel and sexual function. The median follow-up time was 19 months (3-33 months). Quality of life was assessed before radiotherapy, at the end of radiotherapy, three months after radiotherapy, six months after radiotherapy, 12 months after radiotherapy, and 24 months after radiotherapy. The correlation between clinical factors and quality of life was also analyzed using logistic regression. The study showed a transient decrease in urinary tract irritation symptoms or urinary obstruction status scores after radiotherapy (p<0.001); for dyspareunia, bowel reaction and sexual function scores remained stable during the 2-year follow-up. Acute toxicity reactions were 20.3% for grade 1 GU and 10.9% for grade 2 GU; late grade 1 and 2 GU toxicity reactions were 3.1% and 1.6%, respectively. There were no acute and late GI toxicity reactions. Transurethral resection of the prostate decreased risk factors for voiding-related quality of life, age was associated with bowel-related quality of life, and for sexual function status, decompensation status was an important risk factor, and an international prostate symptom score greater than eight increased grade 1 and grade 2 GU toxic reactions. Overall prostate cancer patients treated with carbon ion radiation therapy had a small impact on quality of life.

### Translational Research for Prostate Cancer With Carbon Ion Radiotherapy

**Figure 1:**
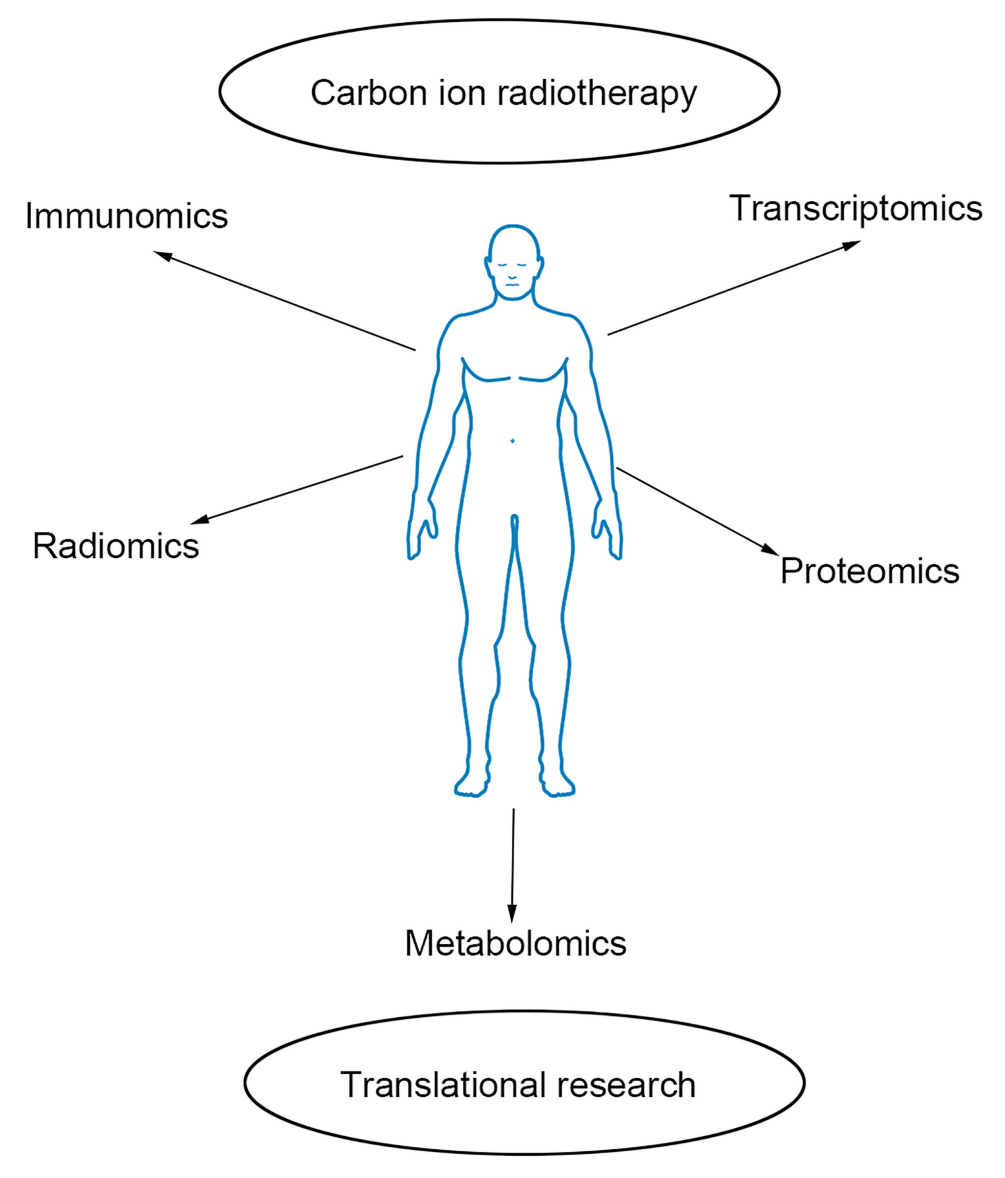
Outline of omics-based translational study. Immunomics, radiomics, transcriptomics, proteomics, and metabolomics are helpful to explore the efficacy and molecular mechanism of prostate cancer with carbon ion radiotherapy.

Carbon ion radiotherapy is the most advanced technique for radiotherapy of prostate cancer. However, few reports discuss the radiobiology of carbon ion radiotherapy and how to assess its efficacy. Our team pays attention to translational research for prostate cancer with carbon ion radiotherapy. We perform omics-based studies such as immunomics, transcriptomics, proteomics, radiomics, and metabolomics to explore prostate cancer’s efficacy and molecular mechanism with CIRT (**Figure 1**).

The immune reaction is relative to the outcome of cancer therapy. However, no reports discussing the immune response in prostate cancer patients with CIRT. We found that lymphocyte subset such as CD3+, CD4+, CD19+, CD4/CD8 ratio predict prostate cancer patients’ short-term efficacy and toxicity with CIRT (54). For another hand, we investigate the association of exosomal miRNA with efficacy. We found that some exosomal miRNAs, especially miR-654-3p and miR-379-5p, may be useful to predict the efficacy of prostate cancer with CIRT (55). Furthermore, we investigated the baseline of MRI radiomics features for prediction of prostate cancer with CIRT; results indicated that radiomics features from T2-weighted images and apparent diffusion coefficient predict the poor and good response of prostate cancer with CIRT (AUC=0.88) (56). Metabolomics is a valuable tool for cancer diagnosis, prediction, and prognosis. Our team investigates the association of metabolic signatures with efficacy and toxicity based on metabolomics, and we verify the metabolic reprogramming of prostate cancer with CIRT. These metabolomics studies are in progress, and we will share the latest research results in the future.

## Discussion

Prostate cancer is a common malignant tumor, and although the incidence in Asia is lower than that in Europe and the United States, the incidence is on the rise and the survival rate is lower than that in Europe and the United States, adding to the burden on society and families (57). Radiotherapy is one of the main treatment modalities for prostate cancer, and photon radiotherapy is currently used in clinical practice (12). Studies have shown that prostate cancer is sensitive to hypofractionated irradiation and conventional photon hypofractionated radiotherapy is beneficial to improve the local control rate of the tumor, but as the dose increases the exposure of the surrounding prostate tissues such as bladder and rectum that endanger the organs is elevated and can easily cause toxic reactions (58). For high-risk prostate cancer, radiotherapy alone is ineffective in controlling biochemical recurrence, and high-dose irradiation combined with endocrine therapy is an effective strategy (41). Conventional photon radiotherapy is also limited by the physical properties, and treating high-risk prostate cancer is also faced with the fact that increasing the dose will increase the toxicity response in normal tissues. Carbon ion radiotherapy has the physical advantage of Bragg’s peak and relatively high biological effect, giving high doses of hypofractionated irradiation to improve the tumor control rate without damaging the endangered organs (18). In Japan, clinical studies of carbon ion radiotherapy for high-risk prostate cancer suggest that carbon ion radiotherapy effectively controls bRFS, OS, and PCSS. The latest results of the Japanese multicenter carbon ion radiotherapy clinical trial showed that the 5-year bRFS, OS, and PCSS for high-risk prostate cancer were 92%, 96%, and 99%, respectively, and the grade 2 GU and GI toxic effects were 4.6% and 0.4%, with no grade 3 or higher toxicity reactions.

In contrast, bRFS and OS for high-risk prostate cancer with conventional photon radiotherapy are lower than carbon ion radiotherapy. For example, James et al. (59) reported a 5-year bRFS and OS of 76% and 90%, respectively, for 180 patients with high-risk prostate cancer treated with 74 Gy irradiation combined with ADT; Valicenti et al. (60) reported a 5-year bRFS of 75% for 66 patients with high-risk prostate cancer treated with 73.8-84.3 Gy irradiation combined with ADT. Germany conducted IPI clinical trials suggesting that the toxicity response of carbon ion therapy was less than that of proton radiation therapy. In Italy, the safety and feasibility of this regimen for prostate cancer treatment were investigated by photon combined with carbon ion irradiation. These studies showed that carbon ion radiotherapy was superior to conventional photon radiotherapy in efficacy control and reduction of toxicity effects. Although there is no clinical phase 3 randomized controlled study comparing the efficacy of carbon ion radiotherapy with photon radiotherapy, the current clinical studies suggest that carbon ion radiotherapy for prostate cancer is worthy of clinical application.

Although carbon ion radiotherapy for prostate cancer reduces treatment outcome heterogeneity, biochemical recurrence still occurs in some patients. Biochemical recurrence may be related to T-stage, initial PSA, risk grade, and duration of endocrine therapy. For high-risk prostate cancer, the optimal duration of endocrine therapy is unclear, and the duration of endocrine therapy is related to the quality of life status. Follow-up randomized controlled studies with large samples are needed to clarify the appropriate duration of neoadjuvant and adjuvant endocrine therapy.

In summary, carbon ion radiotherapy effectively controls prostate cancer progression, has less acute and late toxicity effects, and low impacts on quality of life minimally. The current protocol of 51.2 GyE/12fr. irradiation for 3 weeks is used in Japan to improve the treatment effect while shortening the treatment time. The Shanghai Proton and Heavy Ion Hospital treated prostate cancer with carbon ion radiotherapy and clinical trials are underway. Current follow-up has revealed no significant late gastrointestinal toxicity reactions and no genitourinary toxicity reactions above grade 2. The characteristics of our patient population are different from those of the Japanese population, and currently a 4-week irradiation protocol of 64 GyE/16fr. is mainly used. The transition to a 3-week irradiation protocol will be carried out soon. It is believed that with the accumulation of experience and continuous improvement of technology, carbon ion radiotherapy can be beneficial to more patients.

For translational research, it is important to understand the radiobiology of CIRT and the efficacy of CIRT in prostate cancer patients. Omics-based research provides insight on better view the function of CIRT in prostate cancer. According to the above discussion, it is evidence that CIRT is effective for prostate cancer. Moreover, immunomics, transcriptomics, proteomics, radiomics, and metabolomics can be helpful for individual treatment.

## Conclusions

In conclusion, with physical and biological advantages, carbon ion radiotherapy is effective in prostate cancer. The efficacy response and toxicity response indicate satisfying results in prostate cancer with CIRT. We strongly believe that omics-based studies will become increasingly useful to design individualized treatment strategies.

## Data Availability Statement

The original contributions presented in the study are included in the article/supplementary material. Further inquiries can be directed to the corresponding author.

## Ethics Statement

The studies involving human participants were reviewed and approved by Fudan University Shanghai Cancer center. The patients/participants provided their written informed consent to participate in this study.

## Author Contributions

XC, QY, and PL collected and analyzed data. XC and SF wrote and edited the manuscript. All authors contributed to the article and approved the submitted version.

## Funding

This work was supported by the National Natural Science Foundation of China (No. 81773225).

## Conflict of Interest

The authors declare that the research was conducted in the absence of any commercial or financial relationships that could be construed as a potential conflict of interest.

## Publisher’s Note

All claims expressed in this article are solely those of the authors and do not necessarily represent those of their affiliated organizations, or those of the publisher, the editors and the reviewers. Any product that may be evaluated in this article, or claim that may be made by its manufacturer, is not guaranteed or endorsed by the publisher.
